# *In Vitro* Bactericidal and Bacteriolytic Activity of Ceragenin CSA-13 against Planktonic Cultures and Biofilms of *Streptococcus pneumoniae* and Other Pathogenic Streptococci

**DOI:** 10.1371/journal.pone.0101037

**Published:** 2014-07-09

**Authors:** Miriam Moscoso, María Esteban-Torres, Margarita Menéndez, Ernesto García

**Affiliations:** 1 Centro de Investigaciones Biológicas, CSIC, Madrid, Spain; 2 Departamento de Química-Física Biológica, Instituto Química-Física Rocasolano, CSIC, Madrid, Spain; 3 CIBER de Enfermedades Respiratorias (CIBERES), Mallorca, Illes Balears, Spain; Rockefeller University, United States of America

## Abstract

Ceragenin CSA-13, a cationic steroid, is here reported to show a concentration-dependent bactericidal/bacteriolytic activity against pathogenic streptococci, including multidrug-resistant *Streptococcus pneumoniae*. The autolysis promoted by CSA-13 in pneumococcal cultures appears to be due to the triggering of the major *S. pneumoniae* autolysin LytA, an *N*-acetylmuramoyl-L-alanine amidase. CSA-13 also disintegrated pneumococcal biofilms in a very efficient manner, although at concentrations slightly higher than those required for bactericidal activity on planktonic bacteria. CSA-13 has little hemolytic activity which should allow testing its antibacterial efficacy in animal models.

## Introduction

Despite advances in medicine, infectious diseases remain a major cause of death and inflict social and economic upheaval on millions of people worlwide. Respiratory infections alone are responsible for 4 million deaths every year, with *Streptococcus pneumoniae* the predominant causal agent [1]. Indeed, it is the leading cause of non-invasive infections such as bacterial pneumonia, sinusitis and otitis media (all biofilm-related infections [2]), and is commonly the culprit in life-threatening invasive conditions such as bacteremia/sepsis and meningitis. In developing countries, pneumococcal septicemia is a major cause of infant mortality, leading to the deaths of more than 1.2 million infants every year [3]. HIV infection, sickle-cell anemia, and a variety of chronic organ failure conditions, increase the risk of serious pneumococcal disease.

The alarming, global increase in multidrug resistance, particularly in pneumococcal strains resistant to β-lactams, macrolides, tetracyclines and sulfonamides, has renewed interest in the development of novel drugs and strategies for controlling the spread of antibiotic resistant bacteria [4]. Ceragenins, also known as CSAs (for Cationic Steroid Antibiotics), are based on a cholic acid scaffolding, and mimic antimicrobial peptides [5]. Ceragenins, however, are easier to synthesize than the latter and are much more stable. Further, since they are not peptide-based, they are free from breakdown by proteases, a phenomenon that limits the lifetimes of antimicrobial peptides. Under physiological conditions, ceragenins are polycationic; they therefore associate strongly with anionic bacterial cell surfaces, displacing patches of the membrane [6]. This causes a loss of membrane integrity along with changes in its permeability, eventually leading to the death of affected cells. Ceragenins show strong bactericidal activity against Gram-negative and Gram-positive bacteria (mainly enterococci and staphylococci), and there is little likelihood that natural resistance can be generated against them. It has also been reported that ceragenins potentiate the effect of certain antibiotics on planktonic cells, as well as on mature biofilms produced by a number of *Pseudomonas aeruginosa* strains [7].

**Figure 1:**
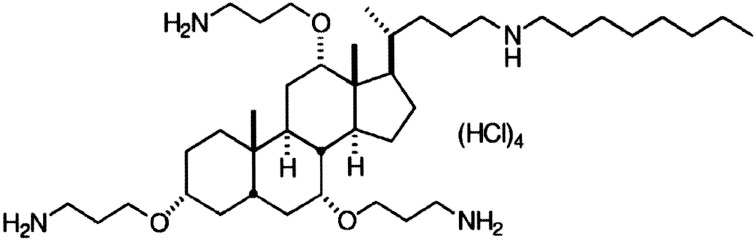
Structure of the ceragenin CSA-13.

The present work examines the antimicrobial activity of the most potent ceragenin, CSA-13 (Fig. 1), against planktonic cultures and biofilms of *S. pneumoniae* and other pathogenic streptococci.

## Materials and Methods

### Ethics statement

The study was submitted to the Consejo Superior de Investigaciones Científicas (CSIC) Ethics Committee which declared it to be carried out with the appropriate ethical safeguards.

### Bacterial strains and growth conditions

The streptococcal strains used in this study are listed in Table 1. These were routinely grown in C medium [8] supplemented (or not) with 0.08% yeast extract (C+Y medium), at 37°C without shaking, or on reconstituted tryptose blood agar base plates (Difco Laboratories) supplemented with 5% defibrinated sheep blood.

**Table 1:** Streptococcal strains used in the present work, and associated CSA-13 MIC values.

Bacterial species/strains^a^	Relevant characteristics(MIC; µg/ml)^b^	CSA-13 susceptibility	Reference/Origin^d^
		(MIC; µg/ml)^c^	
**Mitis group**			
*Streptococcus pneumoniae*			
R6	Unencapsulated laboratory strain, *lytA* ^+^	4 (1)	[57]
P103	Unencapsulated laboratory strain,*lytA*::*aphIII*	4 (1)	This study
Spain^23F^-1	Serotype 23F, *lytA* ^+^(PEN, 1–2; TET, 8; CHL, >8)	4 (1)	[58]
8249	Serotype 19A, *lytA* ^+^(PEN, 6; IPM, 0.35; VAN, 0.4)	4 (1)	[59]
S3	Serotype 23F, Δ*lytA*(PEN, 8; CTX, >16; VAN, 0.5)	8	[60]
SPC2162	Serotype 19A, *lytA* ^+^(PEN, 16)	8	[61]
SPC2552	Serotype 23F, *lytA* ^+^(PEN, 32)	4	[61]
ST942	Non-typeable, *lytA* ^+^	8	[24], [62]
*Streptococcus* sp. strain782/96	*lytA* ^+^(PEN, 0.25; TET, 128; ERY, >128)	(1)	[37]
*Streptococcus* sp. strain11923/96	*lytA* ^+^(PEN, 8; TET, 0.5; ERY, 4)	(1)	[37]
*Streptococcus gordonii* ^T^	Type strain	(2)	CECT
*Streptococcus mitis* NCTC 12161^T^	Type strain	(2)	NCTC
*Streptococcus oralis* NCTC11427^T^	Type strain	(1)	NCTC
*Streptococcus oralis* ^T^(pLSE5)	Type strain, *lytA* ^+^	(1)	[27]
*Streptococcus pseudopneumoniae* CCUG49455^T^	Type strain, *lytA* ^+^	(1)	[63], CCUG
*Streptococcus sanguinis*CECT 480^T^	Type strain	(2)	CECT
**Mutans group**			
*Streptococcus mutans*CECT 479^T^	Type strain	(1)	CECT
**Pyogenic group**			
*Streptococcus agalactiae*CECT 183^T^	Type strain	(1)	CECT
*Streptococcus pyogenes*CECT 985^T^	Type strain	(1)	CECT

a ^T^, type strain.

b CHL, chloramphenicol; CTX, cefotaxime; ERY, erythromycin; IPM, imipenem; PEN, penicillin; TET, tetracycline; VAN, vancomycin.

c MICs in C medium are indicated in parentheses.

d CCUG, Culture Collection, University of Göteborg; CECT, Colección Española de Cultivos Tipo; NCTC, National Collection of Type Cultures.

*Escherichia coli* BL21(DE3) (pMMN1) [9] was grown in Luria-Bertani (LB) medium with ampicillin (100 µg/ml) [10] at 37°C with aeration, as a source of the major pneumococcal autolysin LytA, an *N*-acetylmuramoyl-L-alanine amidase (NAM-amidase; EC 3.5.1.28) [11].

### Transformation procedures

*S. pneumoniae* R6 was transformed with chromosomal DNA from the pneumococcal R924 strain (*lytA*::*aphIII*) [12] by treating precompetent cells with 100 ng/ml of synthetic competence-stimulating peptide 1 (CSP1), as previously described [13]. Transformants were selected by plating in CAT agar supplemented with 3% defibrinated sheep blood, followed by challenge with a 10 ml overlay containing kanamycin (250 µg/ml) [14].

### Antibacterial susceptibility assays

The antibacterial activity of CSA-13, kindly provided by P.B. Savage (Department of Chemistry and Biochemistry, Brigham Young University, Provo, Utah, USA), was determined by standard macrodilution methods according to the Clinical and Laboratory Standards Institute guidelines using an inoculum of ≈4×10^5^ CFU/ml [15]. The MIC was defined as the lowest concentration of CSA-13 that prevented visible growth after 24 h of culture in Mueller-Hinton broth supplemented with 2.5% lysed horse blood (Oxoid), or C medium, at 37°C.

### Time-kill experiments

Unless otherwise stated, exponentially growing cultures of the streptococcal strains were incubated in C+Y medium to an absorbance at 550 nm (*A*_550_) of about 0.2 corresponding to approximately 1–2×10^8^ CFU/ml, depending on the streptococcal strain. CSA-13 was then added at different concentrations to aliquots of the culture, and incubation continued without shaking at 37°C. Growth and lysis were monitored via *A*_550_ values. The viability of a bacterial population was assessed by colony counting on blood agar plates and/or visualized by fluorescence microscopy, using the LIVE/DEAD *Bac*Light bacterial viability kit (Invitrogen-Molecular Probes) to stain cells over a 15 min period in the dark. Bacteria were observed at ×40 and ×100 magnifications using a Leica DM4000B fluorescence microscope equipped with L5 (bandpass 480/40) and N2.1 (bandpass 515–560) filter sets for SYTO 9 and propidium iodide (PI). Both stains are nucleic acid-binding agents but they differ in their spectral characteristics and their ability to penetrate viable bacterial cells. SYTO 9 stains all cells green, while propidium iodide penetrates those with a damaged cell membrane, staining them red.

### Biofilm formation assay

The conditions adopted for biofilm formation by pneumococcal cells and other streptococci in 96-well polystyrene microtiter plates (Costar 3595, Corning Inc.) were those previously described [14]. Cells in late exponential growth were diluted in fresh C medium and about 4.5×10^6^ CFU were dispensed into each well. After 6 h of incubation at 34°C, the biofilm formed was stained with 0.2% crystal violet for 15 min and rinsed to remove non-adherent bacteria. After solubilizing the biofilm in 95% ethanol, the *A*_595_ was determined using an Anthos 2020 microplate absorbance reader (Anthos Labtec Instruments).

For observation of the biofilms by confocal laser scanning microscopy (CLSM), bacteria (about 4.5×10^4^ CFU) were grown on glass-bottom dishes (WillCo-dish, WillCo Wells B.V.) for 12 h at 34°C and then stained with the LIVE/DEAD *Bac*Light bacterial viability kit. The biofilms were observed at ×100 magnification using a Leica TCS-SP2-AOBS-UV CLSM equipped with an argon ion laser. The excitation/emission maxima were around 480/500 and 490/653 nm for SYTO 9 and PI respectively. Images were analyzed using LCS software (Leica). Projections were obtained in the *x*–*y* (scans at 0.5 µm intervals) and *x*–*z* planes (scans at 3 µm intervals).

### Expression and purification of NAM-amidase LytA

*Escherichia coli* BL21(DE3) (pMMN1) was incubated in LB medium containing ampicillin (100 µg/ml) and 0.4 mM isopropyl-β-D-thiogalactopyranoside for the overproduction of LytA, following the previously described protocol [9]. After bacterial disruption in a French pressure cell press, the insoluble fraction was separated by ultracentrifugation (100,000×*g*, 1 h, 4°C), and the supernatant loaded into a DEAE-cellulose column to purify the LytA protein in a single step [16]. The purified enzyme was dialyzed against 20 mM sodium phosphate buffer, pH 6.9, and the protein concentration determined spectrophotometrically.

### Enzymatic assay of LytA activity using radioactively labeled cell walls

Pneumococcal cell walls were radioactively labeled with [*methyl*-^3^H] choline as previously described [17]. Cell wall degradation assays were performed according to standard procedures [18], measuring the amount of radioactivity released into the supernatant. One unit (U) of enzymatic activity was defined as the amount of enzyme needed to release 1 µg (about 700 net cpm) of labeled cell wall material in 10 min at 37°C.

### Hemolytic activity assays

Defibrinated sheep blood (from Biomedics or Oxoid) or human blood from a volunteer (EG) was used in hemolytic activity assays. The blood (5 ml) was centrifuged and the cells washed thoroughly with phosphate-buffered saline (PBS). Cells were collected by centrifugation at 5,000 rpm for 10 min in a Sorvall SS-34 rotor, resuspended in 5 ml of PBS, and stored at 4°C. The number of red blood cells (RBC) was adjusted to ensure total lysis with distilled water. Then, 0.6 ml of RBC suspension in PBS was mixed with 0.6 ml of CSA-13 solution using final drug concentrations proportional to the MIC. The mixtures were incubated at 37°C for 1 h and centrifuged at 800×*g* for 5 min; the supernatant was then removed and the *A*_550_ determined. The percentage of hemolytic activity of the drug at different concentrations was estimated as (*A*−*A*_0_/*A*_max_−*A*_0_)×100, where *A*_0_ is the absorbance associated with the background hemolysis occurring during incubation with PBS, and *A*_max_ the absorbance at 100% hemolysis after incubation in distilled water (for sheep blood) or 0.01% Triton X-100 (for human blood) [19].

### Circular dichroism spectra

Circular dichroism (CD) spectra were recorded at 20°C using a J-810 spectropolarimeter (Jasco Corporation) equipped with a Peltier holder. Far UV spectra were recorded in 0.1 cm-pathlength cells at a protein concentration of 0.16 mg/ml [20]. The observed ellipticities were converted to mean residue ellipticities [θ] assuming a mean molecular mass per residue of 114.8 Da after subtraction of the buffer or the ligand spectra.

### Statistical analyses

The two-tailed Student *t* test, performed using GraphPad InStat version 3.0 software (GraphPad Software, San Diego, CA), was used to determine the differences between means. Data are representative of results obtained from at least three independent experiments.

## Results

### Determination of CSA-13 MICs for the streptococcal strains

Planktonic cultures of eight pneumococcal strains (including the R6 unencapsulated laboratory strain and multidrug-resistant clinical isolates) and of several primary or opportunistic pathogens (Table 1), were tested for their sensitivity to CSA-13. MICs were determined in Mueller-Hinton broth supplemented with 2.5% lysed horse blood, and in C medium in the absence of such blood since some authors have reported that certain antimicrobial agents, such as miltefosine, bind to serum components, reducing the effective concentration [21], [22]. CSA-13 showed potent antimicrobial activity against all the streptococcal strains tested. MICs of 1–2 µg/ml were recorded in the C medium tests, and of up to 8 µg/ml in the lysed blood-containing medium (Table 1).

### Bactericidal and lytic effects of CSA-13 on streptococcal cultures

**Figure 2:**
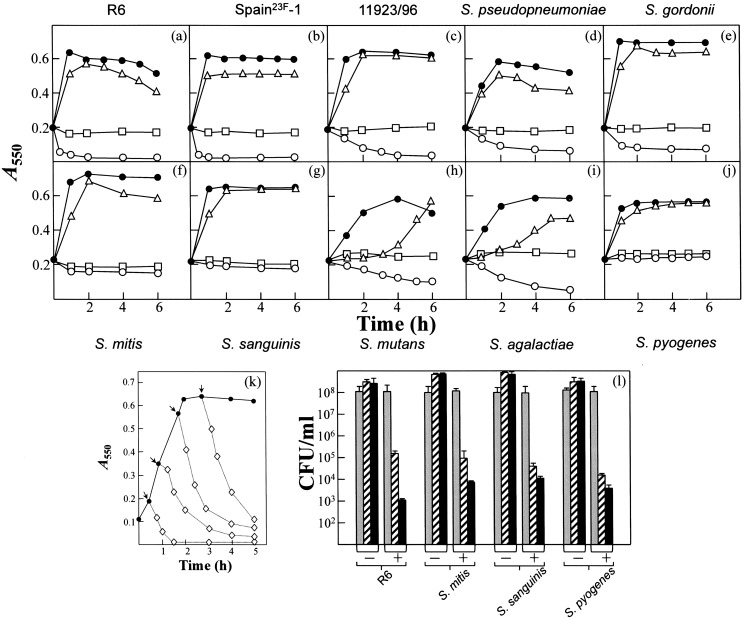
Effect of CSA-13 on streptococcal species. Exponentially growing cultures were incubated in C+Y to an *A*_550_ of about 0.2. CSA-13 was then added to aliquots of the cultures and incubation continued without shaking at 37°C. (a) *S. pneumoniae* R6. (b) *S. pneumoniae* Spain^23F^-1. (c) *Streptococcus* sp. strain 11923/96. (d) *S. pseudopneumoniae*. (e) *S. gordonii*. (f) *S. mitis*. (g) *S. sanguinis*. (h) *S. mutans*. (i) *S. agalactiae*. (j) *S. pyogenes*. Solid circles represent untreated, control cultures. CSA-13 was added (open symbols) at the concentrations: triangles, 1 µg/ml; circles, 10 µg/ml; squares, 25 µg/ml. (k) Lytic effect of CSA-13 (10 µg/ml; open symbols) added to a culture of the pneumococcal strain R6 at the times indicated by the arrows. (l) Bactericidal effect of CSA-13 on different streptococcal species (initial *A*_550_ corresponding to 1–2×10^8^ CFU/ml). Grey, dashed and blackened bars correspond, respectively, to bacterial survival after 0, 2 h, and 6 h incubation in the presence (+) or absence (–) of the ceragenin (10 µg/ml). Standard error bars are shown.

Time-kill experiments were performed with CSA-13 and various streptococcal species. CSA-13 triggered rapid pneumococcal lysis at concentrations of ≥5 µg/ml when added during the early exponential phase of growth (Figs. 2a and b). Interestingly, a lytic effect was recorded in *S. pneumoniae* R6 when the drug was added during the mid-exponential and stationary phases (Fig. 2k). The difference between the MIC and the concentration of ceragenin with *in vitro* activity can be explained in that the efficacy of the antibiotic depends inversely on the initial density of microorganisms [23]. The multidrug-resistant Spain^23F^-1 strain also underwent lysis in the presence of CSA-13, even at concentration of 2.5×MIC (lower than that causing lysis in the R6 strain). Interestingly, *S. pseudopneumoniae* and *Streptococcus* sp. 11923/96, which synthesize a partly active LytA-like autolysin [24], also lysed rapidly in response to CSA-13 (Figs. 2c and d). Other streptococci that underwent lysis after exposure to CSA-13 (10 µg/ml) were *S. gordonii*, *S. mutans* and *S. agalactiae*. Moreover, the growth of *S. mutans* and *S. agalactiae* was slowed at 1 µg/ml of CSA-13, and the cultures lysed with concentrations ranging between 2.5 and 10 µg/ml. *S. mitis*, *S. sanguinis* and *S. pyogenes* stopped growing when treated with CSA-13, but did not lyse (Figs. 2f, g and j). Interestingly, even in the absence of lysis, CSA-13 showed notable bactericidal activity, causing a fall in bacterial survival of up to 4–5-log units after 6 h incubation with 10 µg/ml (Fig. 2l).

**Figure 3:**
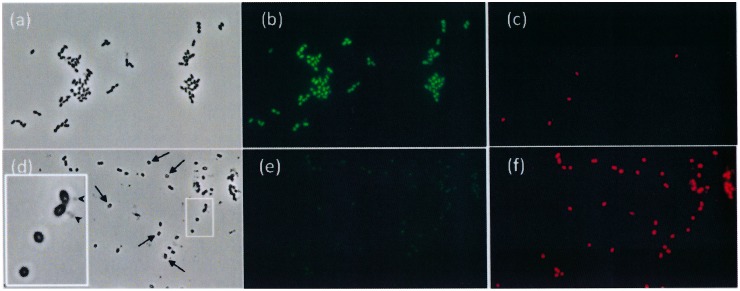
Fluorescence microscopy images of *S. pneumoniae* R6 cells either untreated (panels a–c) or treated with CSA-13 (100 µg/ml; 3 h) (panels d–f) and stained with the *Bac*Light bacterial viability kit. Live and non-viable bacteria fluoresce green (b, e) and red (c, f) respectively. Panels a and d are phase contrast micrographs. The arrows indicate cells treated with CSA-13 and lacking any cytoplasmic content. Panel d (enlarged inset) shows four apparently empty cocci. Some cellular material appears to be undergoing release from two bacteria (arrowheads).

It was noted that bacterial growth stopped but lysis did not occur when pneumococcal cultures were treated with concentrations of CSA-13 ≥25 µg/ml. Nevertheless, micrographs of strains treated with 100 µg/ml CSA-13 showed only non-viable bacteria represented by cocci that apparently lacked intracellular content (Fig. 3d–f).

### CSA-13 triggers LytA activity

**Figure 4:**
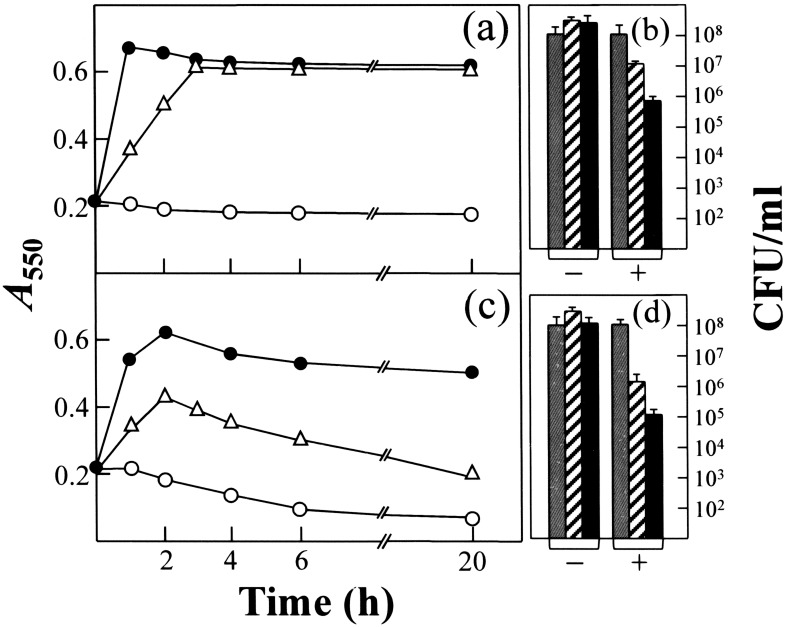
Growth and lysis curves and survival of *S. oralis* (a, b) and *S. oralis* (pLSE5) expressing LytA (c, d) treated with CSA-13. Cells were grown in C+Y at 37°C to an *A*_550_ of about 0.2. CSA-13 was then added to an aliquot of the culture and incubation continued without shaking at 37°C. Solid circles represent untreated control cultures. CSA-13 was added (open symbols) at 2.5 µg/ml (triangles) or 10 µg/ml (circles). For panels b and d, grey, dashed and blackened bars correspond, respectively, to bacteria survival after 0, 2 h, and 6 h incubation in the presence (+) or absence (–) of the ceragenin (10 µg/ml). Standard error bars are shown.

Bile (or sodium deoxycholate) solubility is one of the classic tests for distinguishing *S. pneumoniae* from all other α-hemolytic streptococci [25]. NAM-amidase LytA is responsible for the solubilization of pneumococci by deoxycholate [26]. To determine whether NAM-amidase LytA is involved in the lytic effect of CSA-13, cultures of *S. oralis* (pLSE5) expressing the pneumococcal LytA autolysin [27], and *S. oralis*, which lacks the *lytA* gene, were incubated with CSA-13 in the early exponential phase of growth. *S. oralis* (pLSE5) underwent lysis after exposure to ≥2.5 µg/ml, a response that contrasted to that shown for *S. oralis* (Figs. 4a and c). Moreover, when treated with 10 µg/ml CSA-13 over a 6 h period, *S. oralis* (pLSE5) showed a viable count reduction of 3-logs compared to a 2-log reduction for *S. oralis* (which lacks *lytA*) (Figs. 4b and d). These results suggest that LytA may be responsible for as much as 1-log unit of the cell killing observed.

**Figure 5:**
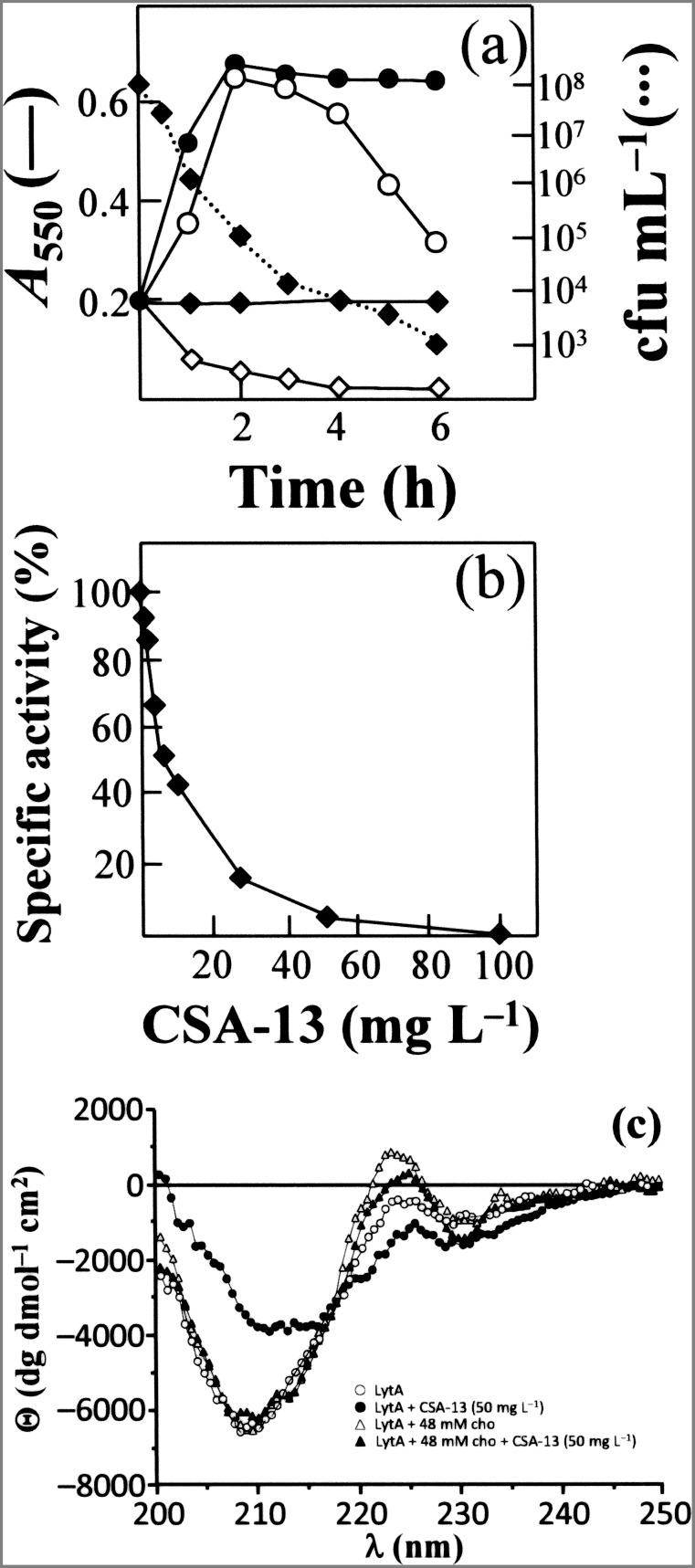
Triggering of the LytA autolysin by CSA-13. (a) An exponentially growing of *S. pneumoniae* strain P103 (*lytA*::*aphIII*) was incubated in C+Y medium with (open symbols) or without (solid symbols) pure LytA enzyme (10 µg/ml) for 30 min at 37°C. The culture was then diluted to an *A*_550_ of 0.2 and divided into two portions. CSA-13 was added at 5 µg/ml (diamonds) to one while the other was left untreated (circles). Incubation was continued at 37°C. Survival of the culture treated only with CSA-13 was determined by plating at different incubation times (dotted line). (b) Effect of CSA-13 on the activity of cell wall hydrolase LytA using radioactively labeled pneumococcal cell walls as substrate. Data represent the percentage activity of LytA in the absence of CSA-13 and are the means of three independent experiments. (c) CD spectra of LytA in the far-UV region in the absence and presence of CSA-13 (50 µg/ml) and/or 48 mM choline chloride (cho).

The *lytA* mutant strain P103, which underwent no lysis in the stationary phase of growth either in the absence or presence of the CSA-13, did undergo lysis after the addition of purified LytA (10 µg/ml) and then CSA-13 (5 µg/ml) (Fig. 5a) –i.e., it was “cured” [28] to the wild-type phenotype. This provides further evidence that the lysis events observed were LytA-mediated. In addition, a rapid loss of viability was observed when the P103 strain was incubated with CSA-13 (a *ca*. 4-log reduction in viable cells after 3 h of treatment), even in the absence of any detectable autolysis (Fig. 5a).

The inhibitory effect of CSA-13 on the lysis of pneumococci when at concentrations ≥25 µg/ml may be due to a direct (or indirect) inhibitory effect of the drug on LytA activity. *In vitro* experiments showed that ceragenin caused a dose-dependent inhibition of NAM-amidase LytA activity (Fig. 5b). A reduction in LytA activity of 85% was observed at 25 µg/ml CSA-13. CD spectroscopy showed that a clear structural alteration of LytA takes place upon incubation with 50 µg/ml CSA-13 (Fig. 5c). In the far-UV region of the LytA spectrum, two negative bands were seen at 210 and 230 nm, a known spectral characteristic [29]. The low absolute value of the ellipticity at 210 nm, and the negative band at 230 nm, reflect a high contribution of aromatic amino acid side chains. Moreover, choline addition to LytA (Fig. 5c) produces a positive band at 224 nm, a red shift in the small negative band at 230 nm, and a slight increase in the negative ellipticity at 210 nm [29]. Interestingly, the changes in the CD spectrum of LytA incubated with CSA-13 were partially reverted by choline chloride, indicating that the ceragenin mainly affects the aromatic acid-rich, C-terminal domain of the NAM-amidase responsible for cell wall binding [11]. These results taken together are in accordance with the inhibition of lysis in pneumococcal cultures treated with high concentrations of CSA-13 (Fig. 2).

### Effect of CSA-13 on biofilm formation by *S. pneumoniae*

**Figure 6:**
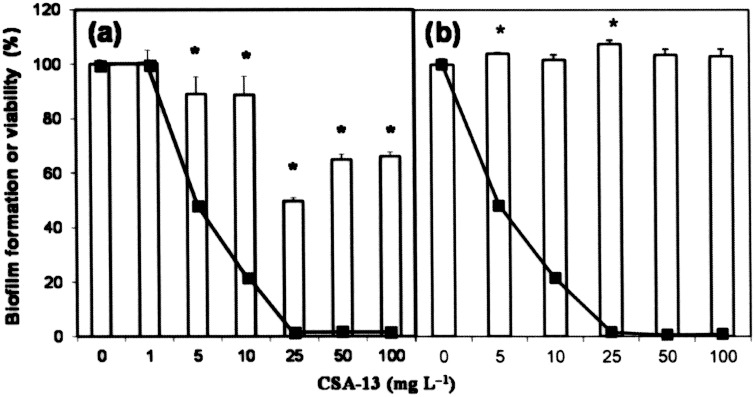
Disaggregation of biofilms in the presence of CSA-13. *S. pneumoniae* R6 (a) and P103 (*lytA*::*aphIII*) (b) were inoculated into 200 µl of C medium in the wells of a microtiter plate (4.5×10^6^ CFU/ml). After biofilm development (6 h at 34°C), CSA-13 was added at different concentrations, and incubation allowed to proceed for 1 h at 34°C before staining with crystal violet to quantify biofilm formation (open bars). Percentage viability (solid lines) after treatment with CSA-13 was determined by plating on blood agar plates. Standard error bars are shown. Asterisks indicate a *P* value<0.05.

**Figure 7:**
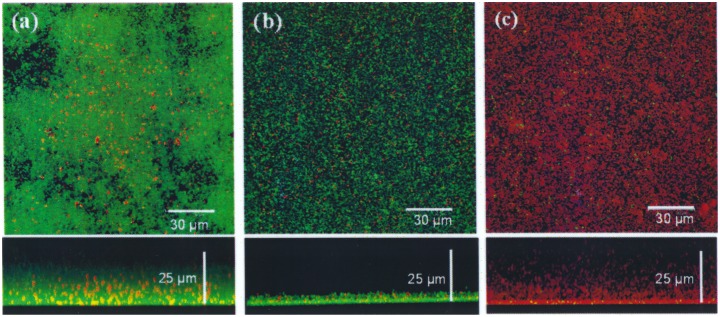
Confocal laser scanning microscopy image of the viability of biofilm-grown *S. pneumoniae* R6 in the presence of CSA-13. Pneumococcal strains (4.5×10^4^ CFU) were grown on glass-bottom dishes (WillCo-dish) in 2 ml of C medium for 12 h at 34°C. The pneumococcal biofilms were rinsed with C medium to remove non-adherent bacteria, and then incubated with CSA-13 at 5 µg/ml (b) or 25 µg/ml (c) for 90 min at 34°C. Panel a shows untreated control biofilm. Cells in the biofilms were stained with the *Bac*Light bacterial viability kit to reveal living (green) and dead (red) bacteria.

To test the capacity of CSA-13 to destroy pneumococcal biofilms, the percentage of biofilm remaining after treatment with different concentrations of CSA-13 above the MIC for 1 h at 34°C was determined. At concentrations of ≥10 µg/ml, CSA-13 effectively disintegrated the biofilms produced by *S. pneumoniae* R6 (Fig. 6a). However, they had no apparent effect on the biofilm produced by the *lytA* mutant P103, as determined by crystal violet staining, although it killed more than 95% of the pneumococci present, as estimated by enumeration of viable counts (Fig. 6b). The viability of pneumococcal biofilms in the presence of CSA-13 was also examined by CLSM (Fig. 7). Biofilms of *S. pneumoniae* R6 grown on glass plates for 12 h at 34°C were treated with CSA-13 (5 or 25 µg/ml) for 90 min and the cells stained using the bacterial viability *Bac*Light kit. A noticeable decrease in biofilm thickness was observed after treatment with 5 µg/ml of CSA-13 (Fig. 7b), and almost all biofilm cells were killed (i.e., they showed red fluorescence) when a dose of 25 µg/ml was used (Fig. 7c).

### Hemolytic activity of CSA-13

CSA-13 showed relatively little hemolytic activity at concentrations equivalent to the MIC in most of the streptococci examined (Table 2). Indeed, less than 10% hemolysis was recorded in human blood at CSA-13 concentrations of ≤10 µg/ml. Total permeabilization of RBC was only achieved with ≥50 µg/ml CSA-13 (not shown). All concentrations of CSA-13 below 50 µg/ml showed less lytic activity against human RBC than against sheep RBC (Table 2).

**Table 2:** Hemolytic activity of CSA-13 on sheep and human blood.

CSA-13(µg/ml)	% Hemolytic activity^a^	
	Sheep blood	Human blood
0.05	7.4	0.0
0.1	8.6	0.0
0.5	13.4	0.1
1	21.1	0.3
2.5	32.9	1.4
5	41.7	3.1
10	52.7	9.0
25	58.7	20.1

a Percentage hemolytic activity estimated as (*A*−*A*_0_/*A*_max_−*A*_0_)×100, where *A*_0_ represents background hemolysis occurring during incubation with PBS, and *A*_max_ the absorbance at 100% hemolysis after incubation in distilled water (for sheep blood) or 0.01% Triton X-100 (for human blood).

## Discussion

*S. pneumoniae* is a major human pathogen and a leading cause of pneumonia, bacteremia and meningitis in adults, and of otitis media in children. Given the rapid dissemination of antibiotic resistance in *S. pneumoniae*, along with other Gram-positive pathogens quoted as “superbugs” [30], new therapeutic strategies are needed to fight the increasing prevalence of multidrug-resistant bacterial infections. The bacterial membrane remains an interesting target since it represents a well-conserved structural element across Gram-positive and indeed Gram-negative bacteria, and because resistance to drugs that attack the membrane would require major –and likely unviable– changes in membrane structure and composition [31]. In fact, CSA-13 resistance in Gram-negative bacteria only correlates with membrane modifications that are unstable in the absence of the drug, and in Gram-positive *Staphylococcus aureus* no CSA-13 resistance has ever been seen, at least under *in vitro* conditions [32].

In this study, we have analyzed the susceptibility of *S. pneumoniae* and other pathogenic streptococci to CSA-13, the most potent member of the ceragenin class. For all the streptococci tested, the MIC values were moderately higher in Mueller-Hinton broth supplemented with 2.5% horse blood than in C medium (Table 1). This is probably due to interaction with serum components reducing the effective concentration of the drug in the former medium. Interestingly, CSA-13 showed comparable activity against the laboratory pneumococcal strain R6, the multi-resistant clinical isolates as strains Spain^23F^-1 and 8249, the vancomycin-tolerant strain S3, and the highly β-lactam-resistant strains SPC2162 and SPC2552 (Table 1). The bactericidal activity of CSA-13 against clinical isolates of resistant *S. aureus* and *P. aeruginosa* strains was reported in earlier studies [31], [33], [34]. The present results show the susceptibility of *S. mutans* and *S. pyogenes* to CSA-13 to be similar to that of *S. mutans* Ingbritt and other clinical isolates of these species [35], [36].

CSA-13 showed a concentration-dependent activity against all the pathogenic streptococci tested. In time-kill analyses, the pneumococcal strains, *S. pseudopneumoniae* and *Streptococcus* sp. 11923/96 underwent autolysis in the presence of 2.5–20 µg/ml CSA-13 (Figs. 2c, d). This is similar to that observed for miltefosine, an alkyllysophospholipid derivative used for the oral treatment of visceral leishmaniasis [22]. These strains synthesize an autolysin closely related to the pneumococcal autolytic NAM-amidase LytA but in contrast with the behavior of the “typical” *S. pneumoniae* autolysin, these enzymes are inhibited by 1% sodium deoxycholate [22], [37]. This lytic effect of CSA-13 was also observed in *S. gordonii*, *S. mutans* and *S. agalactiae*, presumably caused by the triggering of bacterial and/or prophage peptidoglycan hydrolases [38]–[40]. In contrast, other streptococcal species (i.e., *S. mitis, S. sanguinis*, and *S. pyogenes*) did not lyse with CSA-13. However, even when no bacterial lysis was observed, CSA-13 efficiently killed the streptococcal cells (Figs. 2, 4, and 5a).

Undisrupted cocci apparently lacking intracellular content were detected after treatment of pneumococcal cells with 100 µg/ml CSA-13 (Fig. 3d). Similar changes have previously been observed, using transmission electron microscopy, in *E. coli* cells treated with CSAs [41]. Changes indicating wall damage have also been reported in *P. aeruginosa* after treatment with CSA-13, as determined by atomic force microscopy [42]. In agreement with the latter report, CSA-13 was here confirmed to show relatively little hemolytic activity against RBC at concentrations near the MIC for the present strains (Table 2). Cytotoxicity experiments have shown CSA-13 to be mildly toxic to fibroblasts at 100 µg/ml, but not at 75 µg/ml [43]. Interestingly, the cytolytic activity of CSA-13 is greatly reduced in the presence of pluronic acid F-127; consequently, it might be useful for combating streptococcal infections in this combination [34], [42], [44].

The autolysis caused in pneumococcal cultures by CSA-13 (and also in *S. oralis* cultures harboring the plasmid pLSE5 encoding LytA) was due to the triggering of the LytA NAM-amidase, as demonstrated by phenotypic “curing” experiments (Fig. 5a). The rapid lysis of pneumococcal strains (and other streptococcal cultures producing a LytA-like autolysin) is very reminiscent of the lytic response of *S. pneumoniae* to the detergent action of sodium deoxycholate (the bile solubility test) or miltefosine [22], [25], [26]. Since the mid 1970s it has been known that mild detergents, such as sodium deoxycholate, or cell wall inhibitors such a β-lactams, fosfomycin and D-cycloserine, cause the release of lipoteichoic acid from the bacterial cell membrane in a number of streptococci [45], [46]. Pneumococcal lipoteichoic acid is a specific inhibitor of the LytA NAM-amidase [47], and destabilization of the lipoteichoic acid–LytA complex triggers bacterial autolysis [48]. It should be noted that ceragenins and sodium deoxycholate are cholic acid-derivatives, and a similar response to both agents might be anticipated [5]. Importantly, our results showed that CSA-13 possesses bactericidal activity even when no bacteriolysis occurs. This is not completely unexpected since previous work [49] has shown that, although an enzymatically active LytA autolysin is required for β-lactams-promoted lysis (see above), pneumococci are actually killed even though lysis fails to occur.

Bacterial biofilms are implicated in chronic and persistent infections [50] due to the protection they offer against antibiotics and host immune defenses [51]–[53]. Biofilm formation by *S. pneumoniae* on abiotic and biotic substrates (such as adenoid and mucosal epithelia) has been documented [54]. Our results reveal noticeable bactericidal activity by CSA-13 against pneumococcal cells growing in biofilms, a phenomenon associated with the disintegration of biofilm structure (Figs. 6a and 7). However, concentrations slightly higher than those required for bactericidal activity against planktonic bacteria were required to remove pneumococcal biofilms. Recent reports have shown that CSA-13 is effective against biofilms formed by *P. aeruginosa*, *Moraxella catarrhalis*, *Helicobacter pylori*, *S. aureus* and *Enterococcus faecalis* [34], [55], [56].

In summary, CSA-13 is an efficient antimicrobial agent against *S. pneumoniae* and other human streptococcal pathogens *in vitro*; it also destroys pneumococcal biofilms in a very efficient manner. This molecule warrants further investigation as a therapeutic weapon for use against streptococci, particularly multidrug-resistant pneumococci and strains causing biofilm-related infections.
